# Supplementary material for: Supervisors’ ethical leadership and graduate students’ attitudes toward academic misconduct
Source: PLoS One. 2023 Apr 11;18(4):e0283032. doi: 10.1371/journal.pone.0283032 (PMC10089344; doi:10.1371/journal.pone.0283032)
Supplement: Attachment — Submitted filename: Response to Reviewers.docx [file pone.0283032.s002.docx]

**Supervisors’ ethical leadership and graduate students’ attitudes toward academic misconduct**

PONE-D-22-10477

# Summary of the revision

In the last draft, we responded to all your concerns and comments. We solved the weaknesses in theory and methods to make significant improvements. Thank you very much for your recognition of our modification.

Therefore, in this updated draft, we mainly corrected the minor problems. We have modified the notes of Table 1 and Table 2 according to your comments. We have adjusted the format of Figure 1 according to the journal’s requirements. We have checked the writing and grammar.

Thank you again for taking the precious time to help us improve this study.

Best,

The authors' team
